# Novel mutation in ELN gene causes cardiac abnormalities and inguinal hernia: case report

**DOI:** 10.1186/s12887-023-04408-0

**Published:** 2023-11-18

**Authors:** Hua-yong Zhang, Min Xiao, Yong Zhang

**Affiliations:** 1 Department of Cardiology, Wuhan Children’s Hospital (Wuhan Maternal and Child Healthcare Hospital), Tongji Medical College, Huazhong University of Science & Technology, Wuhan, 430016 China; 2 Department of Rheumatology, Wuhan Children’s Hospital (Wuhan Maternal and Child Healthcare Hospital), Tongji Medical College, Huazhong University of Science and Technology, Wuhan, 430016 China

**Keywords:** Supravalvular aortic stenosis, Branch pulmonary artery stenosis, Inguinal hernia, ELN gene, Case report

## Abstract

**Background:**

Elastin-driven genetic diseases are a group of complex diseases driven by elastin protein insufficiency and dominant-negative production of aberrant protein, including supravalvular aortic stenosis (SVAS) and autosomal dominant cutis laxa. Here, a Chinese boy with a novel nonsense mutation in the ELN gene is reported.

**Case presentation:**

We report a 1-year-old boy who presented with exercise intolerance, weight growth restriction with age, a 1-year history of heart murmur, and inguinal hernia. Gene sequencing revealed a novel nonsense mutation in the ELN gene (c.757 C > T (p.Gln253Ter), NM_000501.4). Due to severe branch pulmonary artery stenosis, the reconstruction of the branch pulmonary artery with autologous pericardium was performed. The inguinal hernia repair was performed 3 months postoperatively. After six months of outpatient follow-up, the child recovered well, gained weight with age, and had no special clinical symptoms.

**Conclusion:**

We identified a de novo nonsense mutation in the ELN gene leading to mild SVAS and severe branch pulmonary artery stenosis. A new phenotype of inguinal hernia was also needed to be considered for possible association with the ELN gene. Still, further confirmation will be necessary.

## Background

Elastin is a key protein of the extracellular matrix present in connective tissue, which confers elasticity to the skin, lungs, heart, and blood vessels [1]. Fibers are produced within a limited development window and have an extremely long half-life. The transcript of the elastin gene (ELN, OMIM 130,160) is composed of 34 exons located at 7q11.23 and encodes a series of repeated hydrophobic and crosslinking domains [1, 2]. ELN gene mutations produce diseases by impacting protein quantity and protein quality. Symptoms differ between mutations affecting gene dosage and those producing aberrant proteins in a dominant-negative manner [1].

Mutations within the ELN gene are associated with various elastinopathies, including autosomal dominant cutis laxa (ADCL, OMIM 123,700) and supravalvular aortic stenosis (SVAS, OMIM 18,500) [3, 4]. Although both phenotypes are caused by mutations in the same ELN gene, SVAS and ADCL are mutually exclusive in most patients, suggesting distinct molecular mechanisms [5]. Studies found the majority of ELN gene mutation sites associated with SVAS are normally located in exons 1–29, which leads to insufficient levels of elastin [1, 6]. Similarly, ADCL mutations usually occur in exons 30–34, causing the expression of dominant-negative proteins that interfere with the enzyme’s function [1, 2].

SVAS, characterized by congenital narrowing of the lumen of ascending aorta immediately above the aortic valve, is a rare cardiovascular malformation. Patients with ELN gene mutations are characterized by focal stenosis of the large elastic arteries as the prototypical feature of elastin insufficiency. The stenosis can occur in any elastic artery, but is most common in the supravalvular aortic and branch pulmonary arteries [1, 7, 8]. Further, more distal vessels have been described with narrowing and anatomic abnormalities, including anterior cerebral artery stenosis or aneurysm and coronary artery abnormality. Recent studies suggest that the involved vessels show increased stiffness and may increase the risk of adverse cardiac events. moreover, about 30% of individuals require surgical intervention for the narrow vessels [1, 7–11]. Gastrointestinal features have been described in individuals with ELN gene mutations, including chronic constipation, rectal prolapse, etc. However, the underlying pathology is still not fully known. In this report, we present a boy with a novel mutation in exon 15 of the ELN gene, who presented with mild SVAS, severe branch pulmonary artery stenosis, and inguinal hernia.

## Case presentation

A 1-year-old boy with a 1-year history of heart murmur was referred to our hospital in December 2021. The child was asymptomatic at birth, but a heart murmur was noted at a routine physical examination shortly after birth. Echocardiography showed severe pulmonary artery stenosis and mild SVAS. The child showed exercise intolerance and weight growth restriction with age. Instead, he had no other special clinical symptoms such as syncope, shortness of breath, or cyanosis. On physical examination, a grade 3/6 systolic murmur was heard at the left sternal border. An abdominal examination revealed a right inguinal hernia. The remainder of the physical examination is normal.

In addition, his gross motor, fine motor, personal-social skills, and language skills were commensurate with those of normal peers. Family history revealed that his father and older sister had mild SVAS without clinical symptoms. What’s more, the pulmonary artery flow velocity of his older sister was faster than normal, but it could not meet the diagnostic criteria of pulmonary artery stenosis.

**Fig. 1:**
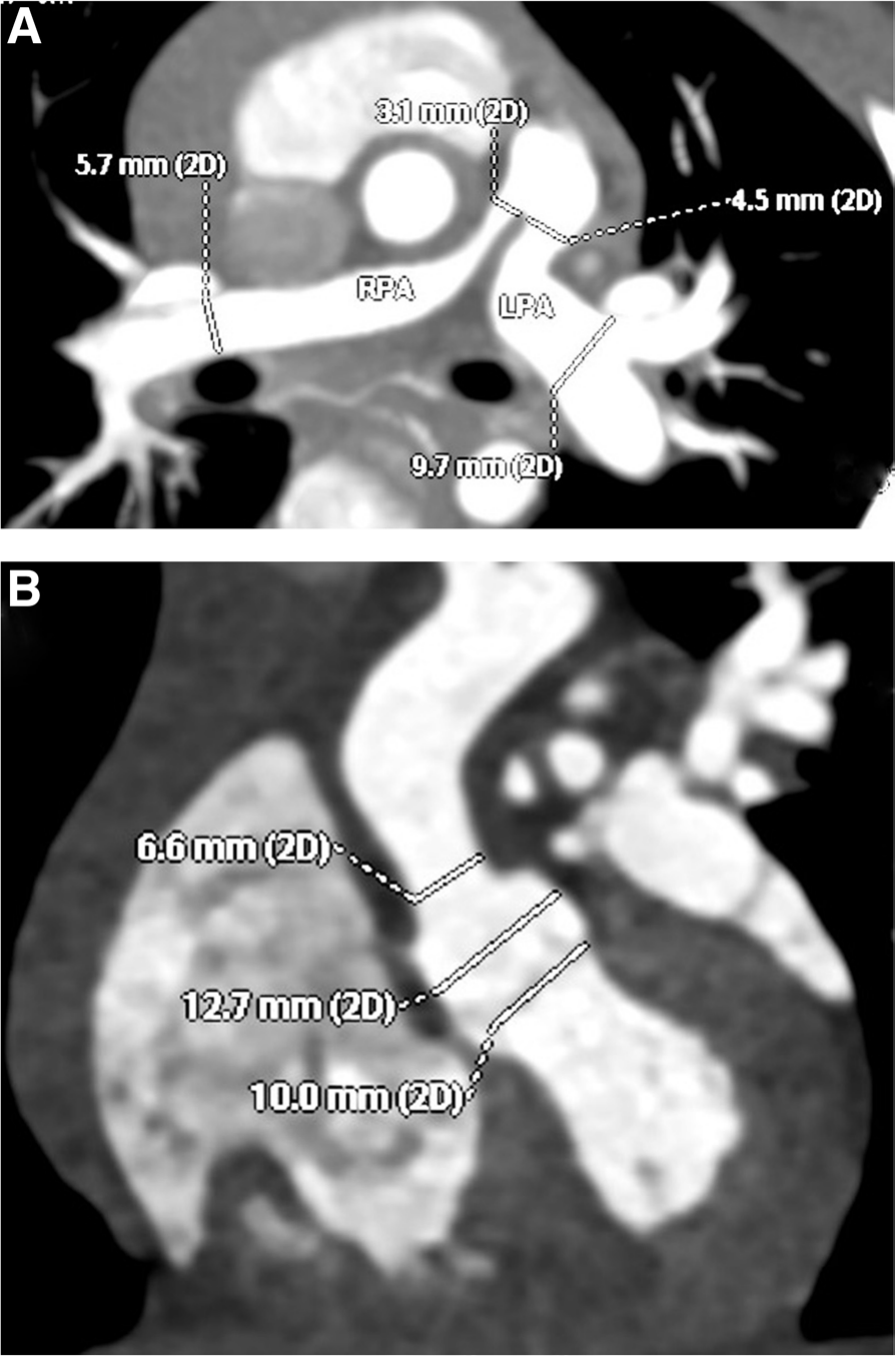
CCTA showed the proximal branch of pulmonary artery stenosis and the aortic wall at the sinotubular junction. **A** The diameter of the main pulmonary artery was 7.1 mm, the diameter of the proximal left pulmonary artery (LPA) was 4.5 mm, the distal bifurcation of the LPA was 9.7 mm, and the proximal right pulmonary artery (RPA) was 3.1 mm, and the distal RPA was 5.7 mm. **B** The diameter of the sinotubular junction was 6.6 mm, the diameter of the aortic annulus was 10 mm, and the diameter of the aortic sinus was 12.7 mm

**Fig. 2:**
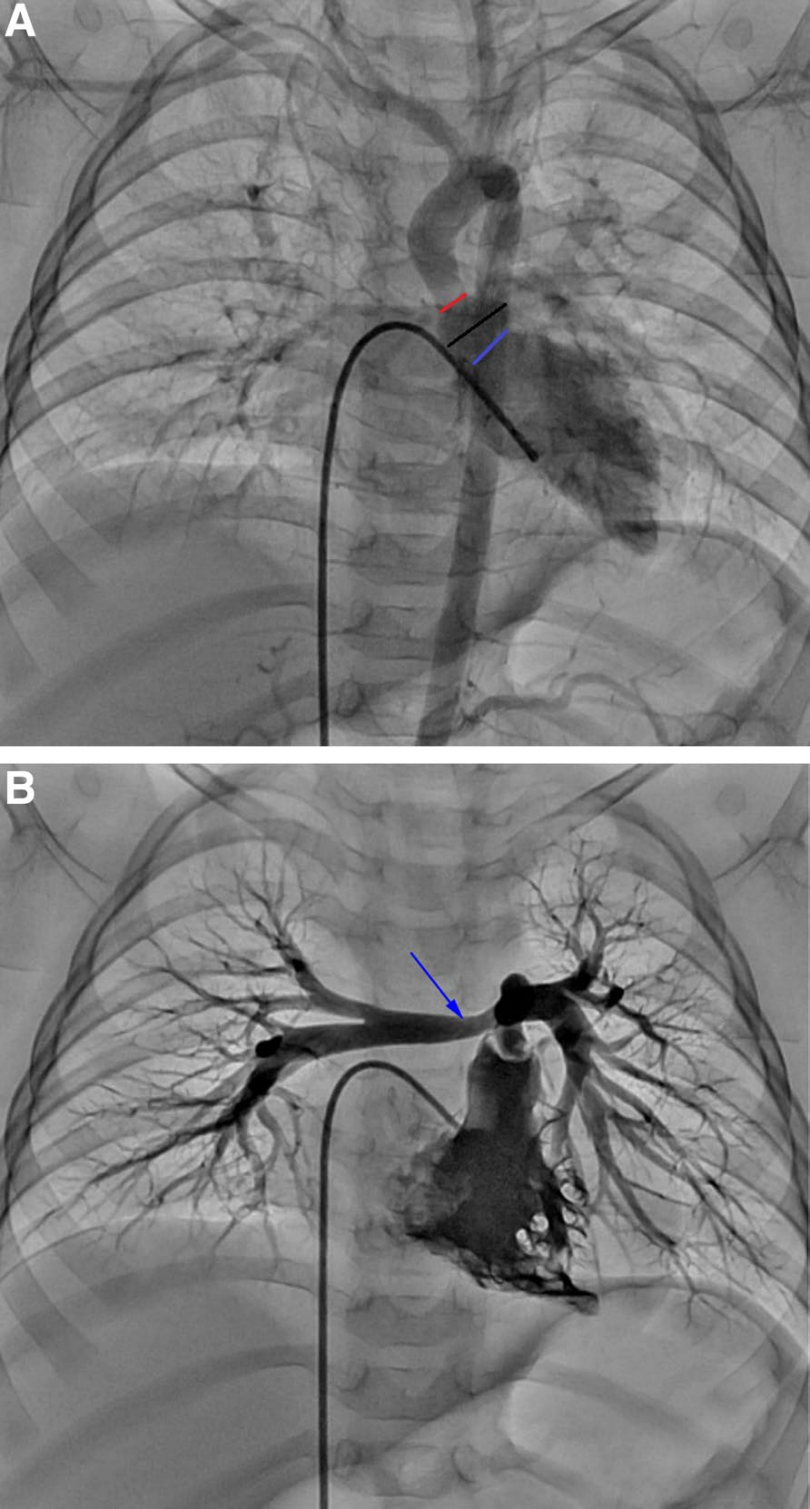
Cardiac catheterization showed the stenosis of the aorta at the sinotubular junction and the branch pulmonary artery stenosis. **A** the sinotubular junction (red line), the aortic sinus (black line), and the aortic annulus (blue line). **B** the severe stenosis of the proximal right pulmonary artery (blue arrow)

Echocardiographic examination was performed after admission to the hospital, which showed severe pulmonary artery stenosis (pressure gradients (PG) between the right ventricle and pulmonary artery = 75mmHg), and the thickening and stenosis of the aortic wall at the sinotubular junction (diameter = 6.8 mm, normal range 9 ~ 13.5 mm). Cardiac computed tomographic angiography (CCTA) showed the proximal branch of pulmonary artery stenosis (shown in Fig. 1A), and the stenosis of the aorta at the sinotubular junction (shown in Fig. 1B). Cardiac catheterization was performed and indicated mild sinotubular junction stenosis with a pressure gradient of 15mmHg (shown in Fig. 2A), the branch pulmonary artery stenosis (PG = 84mmHg) (shown in Fig. 2B).

**Fig. 3:**
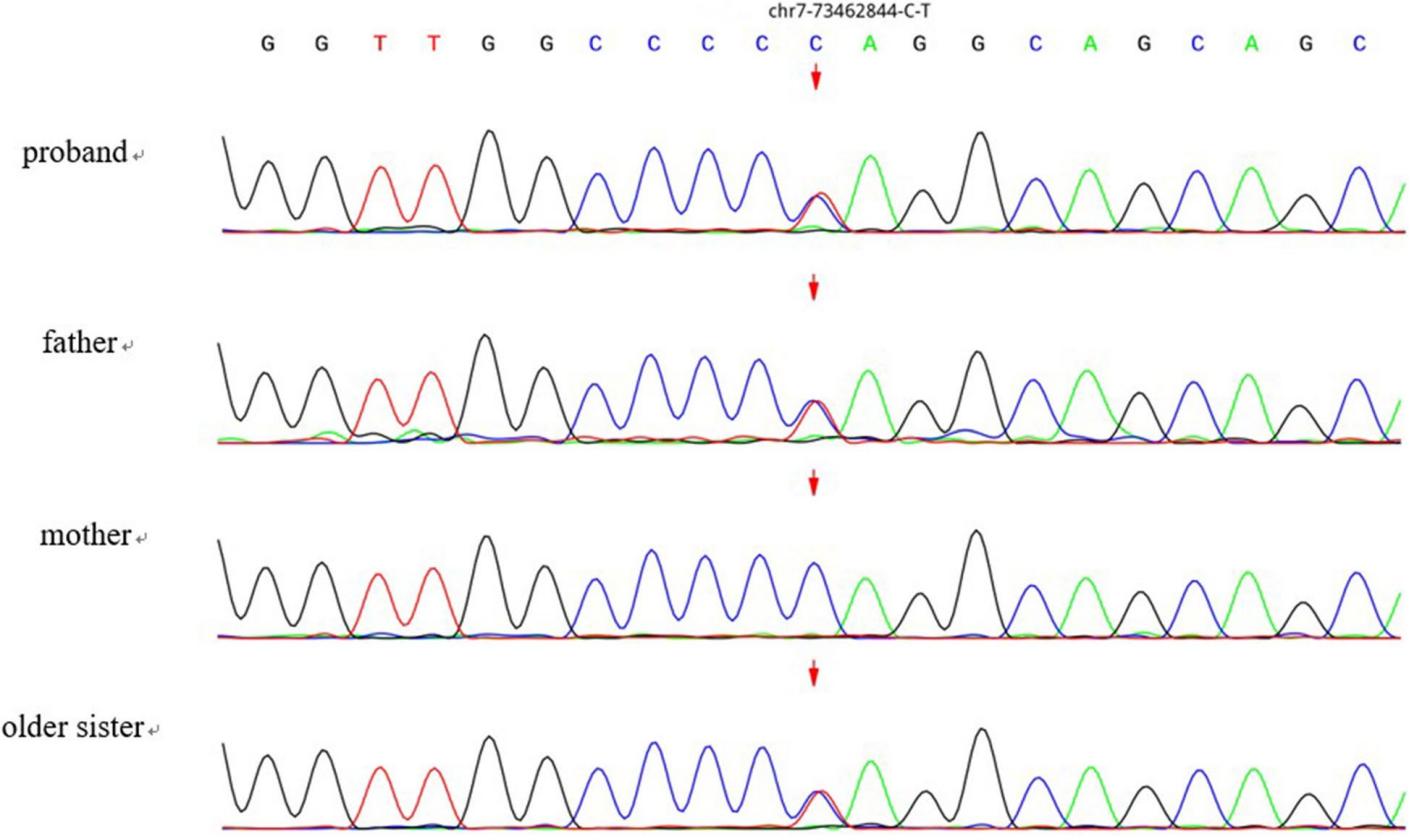
Whole-exome sequencing identified mutation in the proband, his older sister, and his parents. The identified point mutation in exon 15 of the ELN, generated a stop codon (c.757 C > T (p.Gln253Ter), NM_000501.4)

Peripheral blood genomic DNA from the patient, his older sister, and his parents was used for whole-exome sequencing (WES). A novel nonsense variant c.757 C > T (p.Gln253Ter) in ELN was identified in the proband (shown in Fig. 3), which was not previously reported in the database of the Human Gene Mutation Database, ClinVar variation Database, or previous literature. His father and older sister were verified as ELN heterozygous. This variant was located in exon 15, which changed glutamic acid to a premature termination codon, leading to a truncated protein. American College of Medical Genetics and Genomics (ACMG) guidelines and standards describe this variant as probably pathogenic.

After completing all the tests mentioned above, we found that the ELN gene causes mild SVAS and severe branch pulmonary artery stenosis in this patient. Due to severe branch pulmonary artery stenosis, the reconstruction of the branch pulmonary artery with autologous pericardium was performed. In 8 days following the operation, the patient was discharged from the hospital without experiencing any discomfort. Moreover, the right inguinal hernia repair was performed 3 months postoperatively. Reexamination of echocardiography was performed at 1-month, 3-month, and 6 months after surgery, which indicated the pulmonary artery flow velocity was lower than 2 m/s. meanwhile, the child gained weight with age and had no special clinical symptoms such as exercise intolerance, syncope, shortness of breath, or cyanosis.

## Discussion and conclusion

This report describes a patient who presented with mild SVAS, severe branch pulmonary artery stenosis, and an inguinal hernia due to a novel variant of the ELN gene (NM_000501.4: c.757 C > T, p.Gln253Ter). This variant was located in exon 15, which changed glutamic acid to a premature termination codon, leading to a truncated protein. More than 100 variants of ELN have been identified in literature so far as pathogenic or possible pathogenic [1]. Variants causing the SVAS and branch pulmonary artery stenosis phenotypes are associated with decreased total elastin levels, which would likely induce nonsense mediated mRNA decay, and lead to ELN haploinsufficiency [1, 2, 6, 9]. Interestingly, the proband’s father and older sister displayed a milder phenotype despite the presence of the same ELN gene variation. The clinical heterogeneity might indicate a partly incomplete penetrance. Compared with this study, the previous study reported that the c.757delC frameshift mutation can also lead to the similar phenotype [1]. Deletion of the whole ELN gene can potentially result in SVAS in addition to these single base pairs or brief variants [12]. For instance, the Williams critical region, located in 7q11.23, including the ELN gene and neighboring 25–27 genes, is commonly deleted in Williams syndrome (WBS) [1, 13, 14]. WBS shares certain vascular characteristics with SVAS, but the symptoms are more complex due to the loss of other genes [4, 10, 14, 15].

Elastic lamellae and fibers are arranged circumferentially in the vasculature, sandwiched between the smooth muscles. Studies showed that smooth muscle and elastic lamellae increased in mice and humans with insufficient elastin arteries. however, the content of elastin in each layer has markedly decreased [1, 16, 17]. When the vessel wall changes, the biomechanical properties change as well, resulting in a vessel with a thicker wall, smaller lumen, and decreased compliance [18, 19]. Human ELN gene variants cause elastin insufficiency characterized by focal narrowing of the large elastic arteries, particularly the ascending aorta and branch pulmonary arteries [1, 7]. There have been reports of abnormalities in other vessels besides the aortic and pulmonary arteries, including coronary artery abnormality, cerebral artery stenosis or aneurysm, and renal artery stenosis [1, 9, 20]. Stroke and sudden death due to the abnormalities of the vasculature have been reported in individuals with isolated SVAS and WBS [1, 9, 20]. According to studies, approximately 30% of cases with stenosis needed surgical intervention, and about 20% showed no significant stenosis [1]. In this study, the branch pulmonary artery of the individual was reconstructed with the autologous pericardium, which seemed to have a favorable prognosis. Simultaneously, no immediate surgical intervention for the mild SVAS was required. There is, however, an interesting point to remark that the SVAS may be aggravated with time [1]. Therefore, continuous follow-up is required for judgment of prognosis in the long term.

The underlying pathology of inguinal hernia is still not fully known. Genetic factors may play an interesting role [1]. Previous study indicated a possible association between the ELN gene and inguinal hernia. The decreased expression of the ELN gene in the transversalis fascia may lead to the formation of inguinal hernia [21]. In the present study, the clinical phenotype of inguinal hernia has also been noticed. However, it would require further validation.

Currently, in elastin insufficiency, investigational treatments have focused on increasing elastin production and reducing smooth muscle proliferation. There are, however, no FDA-approved treatments that target the pathogenesis of the disease [1]. Therefore, in addition to surgical intervention, further studies for individuals with ELN gene mutations to improve outcomes are warranted.

In conclusion, we identified a de novo nonsense mutation in the ELN gene leading to mild SVAS and severe branch pulmonary artery stenosis. A new phenotype of inguinal hernia was also needed to be considered for possible association with the ELN gene. Still, further confirmation will be necessary.

## Data Availability

The data used for this case report is available upon reasonable request.

## Abbreviations

SVAS: Supravalvular aortic stenosis
ADCL: Autosomal dominant cutis laxa
PG: Pressure gradients
CCTA: Cardiac computed tomographic angiography
WES: Whole-exome sequencing
ACMG: American College of Medical Genetics and Genomics
